# Early maladaptive schemas, distress tolerance and self-injury in Iranian adolescents: serial mediation model of transdiagnostic factors

**DOI:** 10.1192/bjo.2024.708

**Published:** 2024-05-21

**Authors:** Maryam Babaeifard, Mehdi Akbari, Shahram Mohammadkhani, Jafar Hasani, Reza Shahbazian, Edward A. Selby

**Affiliations:** Department of Clinical Psychology, Faculty of Psychology and Education, Kharazmi University, Tehran, Iran; Department of Computer Engineering, Modelling, Electronics and Systems Engineering, University of Calabria, Rende, Italy; Department of Psychology, Rutgers University, New Brunswick, New Jersey, USA; Department of Clinical Psychology, Faculty of Psychology and Education, Kharazmi University, No. 43, South Mofatteh Ave., Tehran, Iran

**Keywords:** Non-suicidal self-injury, adolescents, early maladaptive schemas, distress tolerance, experiential avoidance

## Abstract

**Background:**

Non-suicidal self-injury (NSSI) is prevalent behaviour among adolescents. Although there are different etiological models of NSSI, there is a general lack of evidence-based, comprehensive and transdiagnostic models of NSSI in adolescents.

**Aims:**

The aim of this study was to investigate a model of transdiagnostic factors of NSSI in adolescents, testing a serial mediation model of the relationship between early maladaptive schemas (EMS), distress tolerance and NSSI through experiential avoidance and rumination.

**Method:**

A community sample was identified of 1014 adolescents aged 13–17, of whom 425 had a history of NSSI. A serial mediation path analytic method was utilised to examine the relationships between NSSI and its associated functions as criterion variables, EMS and distress tolerance as predictors, experiential avoidance as the first mediator and rumination as the second mediator.

**Results:**

The path analytic model fit indices were good (X^2^/d.f. = 2.25, goodness of fit index = 0.98, normed fit index = 0.97, comparative fit index = 0.98, root mean square error of approximation = 0.054, standardised root mean squared residual = 0.028). Rumination significantly mediated the relationship between schemas of ‘vulnerability to harm’, ‘emotional deprivation’, ‘social isolation’, ‘insufficient self-control’, and NSSI frequency and intrapersonal functions. In serial fashion, experiential avoidance mediated the role of rumination in the relationship between social isolation, and insufficient self-control and NSSI frequency and intrapersonal functions. All indirect effects were significant.

**Conclusions:**

Key indirect effects were found linking maladaptive schemas and distress tolerance to NSSI frequency, and NSSI intrapersonal functions via experiential avoidance and rumination. Thus, it is important to address these transdiagnostic factors with particular emphasis on the sequential mediating role of experiential avoidance and rumination in conceptualisation and therapeutic interventions for NSSI.

Non-suicidal self-injury (NSSI) is defined as direct and deliberate destruction of one's own bodily tissue in the absence of suicidal intent.^1^ NSSI prevalence rates vary from 5.5% among adults to 6.2% among preadolescents,^2^ 17.2% among adolescents and 13.4% among young adults.^3^ Examining NSSI in community samples of adolescents is particularly important because it has been shown that adolescents who engage in NSSI in community samples frequently do not seek help.^4^ This can indicate the importance of identifying the key cognitive processes in conceptualisation and innovative interventions for this behaviour in adolescents. Such processes include emotion regulation deficits,^1^ distress tolerance^5^ and interpersonal motivations that serve as a function for NSSI.

## Adolescence and risk for NSSI

Although NSSI is a serious behaviour that can continue well into adulthood, the highest rates of the behaviour are seen in adolescents.^2^ The adolescent years are filled with numerous internal challenges including identity, social connection, physical development and schema formation for concepts of self and others. In addition, there are several external challenges including fulfilling family and community expectations, potential bullying and growing challenges with social media. Coping with these challenges is often stressful, and NSSI likely emerges in adolescence as a form of emotion regulation that youth use to cope with such stressors. Thus, improving our understanding of adolescent experiences with cognitive scheme development, distress tolerance and emotion regulation can help us understand why NSSI is so fundamentally linked to the adolescent developmental stage.

## Models of emotion regulation for NSSI

Up to now there have been different emotion regulation-focused models that try to explain the underlying components related to NSSI. For example, according to the process model of emotion regulation developed by Gross,^6^ emotions are elicited following an internal or external situation in which attention is focused on specific characteristics that are appraised by the individual, and this leads to a response. This model comprises five stages: (a) selecting situation, (b) modifying situation, (c) deploying attention, (d) changing cognitive processes and (e) modulating response.^6^ This process model of emotion regulation has been applied to explain how NSSI can be conceptualised as a strategy of emotion regulation in any of these stages in the model.^7,8^ For example, in the situation selection stage, NSSI can be used as a way of avoiding aversive situations. NSSI can also be considered as a way of modulating the situation by evoking a response from others. In the attentional deployment stage, NSSI can be a distraction from aversive emotions.^9^ Self-injury can even be a form of cognitive change, because it leads to relieve guilt.^8^ In addition, previous research showed that cognitive reappraisal was negatively correlated with NSSI.^10^ Finally, this behaviour is said to be applied as a way of reducing distress or aversive emotional responses, and as research has found, expressive suppression has been shown to be positively correlated with NSSI among adolescents.^11^

The difficulties in the emotional regulation model^12^ focus on deficiencies in the ability to experience emotions. This model contains some essential core concepts: (a) being aware of emotions, (b) accepting emotions, (c) being able to control impulses consistent with goals, and (d) being flexible in various contexts. In a meta-analytic study, it was found that higher levels of emotional dysregulation in all dimensions, including lack of emotional awareness, lack of emotional clarity, non-acceptance of emotional responses, limited access to effective emotional regulation strategies, difficulties controlling impulses and difficulties engaging in goal-directed behaviours, were linked with an increased risk of NSSI.^13^ In addition, it has been shown that NSSI is related to difficulties with emotion regulation together with each of the subscales of the Difficulties with Emotion Regulation Scale.^12^

A model that has been particularly developed to conceptualise NSSI is the experiential avoidance model,^14^a behavioural model that focuses on the fact that NSSI is used as a way of avoiding aversive and unwanted emotions. This phenomenon is known as emotional avoidance. The model also emphasises several factors including emotional intensity, deficits in emotion regulation skills and distress intolerance that may contribute to experiential avoidance. Recently a systematic review study has been conducted on the relationship between experiential avoidance and NSSI, according which there is a robust link between them.^15^ It has also been shown that in community-based samples, the frequency of NSSI is linked to experiential avoidance.^16^

Key cognitive processes have also been implicated in NSSI. The Emotional Cascade Model (ECM1-6) has been based on Linehan's work^17^ with borderline personality disorder. Emotional cascades are the core of the model that can enhance the risk of engaging in dysregulated behaviours such as NSSI via a cycle of rumination. Rumination is a mode of responding to distress that the individual engages in, repetitively and passively focused on the symptoms of distress.^18^ A systematic review study has also demonstrated the link between rumination and NSSI, reported by various studies.^19^

More recent is the cognitive–emotional model of NSSI, whose main focus is on cognitive processes and their interaction with emotion regulation.^20^ This model utilises social cognitive theory in order to integrate models of emotion and models of cognition for conceptualising NSSI. The model has also been applied in a sample of university students.^21^

## Common points and critique of models

All the models described have some common components and specific characteristics. The first common point is that all models focus on a trigger or situation that evokes emotions. Then all models mention the presence of emotional experience. The other common point is the role of cognitive processes, particularly in ECM1^22^ with emphasis on rumination and cognitive reappraisal in Gross's model. However, because of the lack of attention to cognitive processes in the former models, excluding the cognitive emotional model of NSSI,^20^ the expanded role of cognition has been thoroughly explored, or the importance of verbal cognitions or thoughts and beliefs about NSSI has been considered.

In enhancing our understanding of the cognitive factors involved in NSSI, there are some gaps that should be addressed. For example, it is not yet clear whether these cognitive models are applicable for community-based samples of adolescents. In addition, despite the fact that some constructs related to childhood experiences like early maladaptive schemas (EMS) play an important role in NSSI,^23^ they have not been well addressed in the models of NSSI. Furthermore, because of the transdiagnostic nature of NSSI,^24^ the role of transdiagnostic factors and their relationship in the development and maintenance of NSSI has not been identified, and little is known about important factors that may have a role in NSSI engagement. Therefore, it seems important to explore models that can start to address some of these gaps.

## Towards exploring the transdiagnostic factors of NSSI in adolescents

Numerous studies have shown that NSSI and its functions are associated with EMS,^23^ defined as dysfunctional pervasive patterns that consist of emotions, cognitions, memories and somatic-based elements about oneself and one's relationship with others.^25^ In addition, with regard to the link between EMS and NSSI in adolescents, it has been indicated that schemas of emotional deprivation, vulnerability to harm, subjugation and self-sacrifice can predict NSSI in adolescents.^26^

Another factor that has been shown to be correlated with NSSI is distress tolerance. Studies have shown the link between distress tolerance and NSSI.^27,28^ Based on the experiential avoidance model, when a stimulus recalls aversive experiences, including emotions and thoughts, and somatic sensations, low tolerance may increase the urge to engage in NSSI as a way of getting rid of such experiences;^14^ in fact, low distress tolerance may lead to NSSI via experiential avoidance. In addition, it seems that individuals with low distress tolerance use NSSI as a way of escaping from aversive emotions, which indicates NSSI intrapersonal function. It has also been shown that distress tolerance and rumination interact to predict the odds of NSSI;^5^ however, the exact mechanism is not yet clear. It seems that low distress tolerance may lead to NSSI via engaging in rumination.

The role of avoiding aversive emotions in NSSI engagement has been emphasised in the model of experiential avoidance.^14^ In fact, it may be that an individual with some particular EMS tries to avoid experiencing emotions evoked by schemas, and chooses to self-injure in order to release the pain. It seems that experiential avoidance can play a mediating role in the relationship between schemas and NSSI. In addition, research has indicated that individuals with a history of aversive experiences, leading to subsequent development of EMS, are more predisposed to involve in rumination.^29^ On the one hand, research has suggested that rumination is associated with engaging in NSSI,^19^ and as discussed above, according to the Emotional Cascade model, a cycle of rumination can associate the relationship between the emotional cascades and NSSI.^22^ In the same way, encountering the aversive emotions evoked by schemas, the individual engages in NSSI via rumination. So, it seems that rumination may also have a mediating role in the relationship between EMS and NSSI. On the other hand, there are some studies that have reported a relationship between experiential avoidance and depressive rumination,^30^ so it may be that there is also a link between experiential avoidance and rumination that can sequentially mediate the relationship between EMS with NSSI.

With regard to NSSI functions and their relationship with the mentioned constructs, especially the link between EMS and NSSI interpersonal and intrapersonal functions through rumination, and in order to better understand the path from schemas to the functions through potential mediating variables, we categorised the schemas into interpersonal and intrapersonal schemas in a similar way to that in the research of Quirk et al,^29^ which created two categories of schemas: intrapersonal schemas that are considered to be self-focused, and interpersonal schemas that are other-focused.

In addition, several studies have shown that impulsivity and childhood traumatic experiences are strongly correlated with NSSI.^31,32^ So, in order to better determine the role of other constructs in the context of NSSI engagement, impulsivity and childhood traumatic experiences were controlled in this study.

## Current study

Taken together, it seems that all the constructs mentioned above play a role in NSSI engagement. On the one hand and as discussed earlier, the association between EMS and distress tolerance with NSSI has been identified in different studies^27,28^. On the other hand, it may be that these constructs are associated with NSSI and its functions via experiential avoidance and rumination sequentialy. Despite the fact that all these variables have been identified in different studies, the underlying relationship between them is not yet clear. In fact, the important transdiagnostic factors that can shed light on the conceptualisation of NSSI engagement and its functions for adolescents were considered separately through different models, but the role of these transdiagnostic factors and potential mediating effects have not been addressed in a unified model for adolescents. In addition, given that childhood traumatic experiences and impulsivity have been shown to play an important role in NSSI engagement^31,32^, we controlled them in this study. Thus, the present study aimed to investigate the relationship between EMS and NSSI, as well as the link between distress tolerance and NSSI and its functions, controlling for childhood traumatic experiences and impulsivity. Furthermore, it was hypothesised that these associations are mediated by rumination and experiential avoidance, and it is also expected that experiential avoidance and rumination can act as mediators sequentialy.

## Method

### Participants and procedure

The participants in this study were recruited from ten high schools in Tehran Province, Iran. The schools were randomly selected from the list of available schools. Using the ratio of the observations to estimated parameters as a guide,^33^ we considered 20 observations for each parameter. The initial participants were 1014 adolescents. Of these, 78 adolescents did not complete all the questionnaires, and 511 adolescents (55% females) aged from 13 to 17 (*M =* 15.52, s.d. = 1.30) reported that they had never engaged in NSSI, so they were not included in the final sample. Therefore, the final sample consisted of 425 adolescents (51.8% female) aged 13 to 17 (*M =* 15.60, s.d. = 1.19), of which 5.2% were 7th grade students, 19.9% were 8th grade students, 24.2%were 9th grade students, 22.5% were 10th grade students and 28.1% were 11th grade students. In addition, all students were unmarried and unemployed. There were no missing data. Consent was obtained from the participants and their parents. In addition, participants were assured that the questionnaires and their responses were completely confidential and anonymous. All participants completed the questionnaires simultaneously, and in order to prevent cheating, the research team were present and watched the students carefully.

## Ethics statement

The authors followed all ethical guidelines with the human subjects who participated in the research, including provision of informed consent. The procedure was carried out in accordance with the ethical standards of the Ethics Committee of Kharazmi University of Tehran, Iran (IR.KHU.REC.1401.054) and with the Declaration of Helsinki.

### Measures

#### Young Schema Questionnaire-Short Form

The Young Schema Questionnaire-Short Form (YSQ-SF^34^) is a self-report measure with 75 items developed to assess 15 different maladaptive schemas. In the final model we utilised four schemas: emotional deprivation (e.g. ‘For much of my life, I haven't felt that I am special to someone’*)*, social isolation (e.g. ‘I don't belong; I'm a loner’), vulnerability to harm (e.g. ‘I worry about being attacked’) and insufficient self-control (e.g. ‘I have rarely been able to stick to my resolutions’). The scale is rated from 1 (completely untrue of me) to 6 (describes me perfectly). The psychometric properties of the short form of this instrument also appear to be on par with those of the full (205-item) scale, demonstrating similar levels of reliability, validity and clinical utility.^35,36^ This scale has been validated in Iran, and the findings have shown a good reliability for subscales, α = 0.62–0.90.^37^ The internal reliability of the YSQ-SF in this study was 0.95.

#### Distress Tolerance Scale

The Distress Tolerance Scale (DTS^38^) is a self-report measure with 15 items that assess one's ability to tolerate emotional distress. DTS has four subscales: (a) tolerance (e.g. ‘I can't handle feeling distressed or upset’), (b) appraisal (e.g. ‘My feelings of distress or being upset are not acceptable’), (c) absorption (e.g. ‘When I feel distressed or upset, all I can think about is how bad I feel’) and (d) regulation (e.g. ‘I'll do anything to avoid feeling distressed or upset’). Higher scores indicate a greater ability to tolerate distress. The total DTS and its subscales showed good internal consistency, convergent and divergent validity, and adequate test–retest.^38^ The measure has been validated in Iran, and the scale reliability has been reported at 0.67.^39^ The scale reliability was good in the current sample, α = 0.80.

#### Ruminative Response Scale

The Ruminative Response Scale (RRS^40^) is a self-report measure developed to evaluate the tendency to respond to depressed moods. The RRS is composed of 22 items, all rated on a 4-point Likert-type scale, ranging from 1 (almost never) to 4 (almost always). Treynor and Gonzalez^41^ identified two main components for rumination: reflection (e.g. ‘Write down what you are thinking and analyse it’) and brooding (e.g. ‘Think ‘Why can't I handle things better?’’), and reported that the other 12 items are depression related (e.g. ‘Think about how alone you feel’). The RRS has been shown to be a reliable and valid measure of rumination, and Cronbach's alpha has been shown to be above 0.90.^42^ In Iran, this scale has been validated, and the findings showed that the Cronbach's alpha was 0.90.^43^ In this study the full RRS score was utilised; Cronbach's alpha was 0.87 in this sample.

#### Avoidance and Fusion Questionnaire for Youth

The Avoidance and Fusion Questionnaire for Youth (AFQ-Y8^44^) is the short version of the Fusion Questionnaire that includes eight items (e.g. ‘My thoughts and feelings mess up my life’; ‘The bad things I think about myself must be true’). The questionnaire is rated on a 5-point scale ranging from 0 (not at all true) to 4 (very true). Higher scores indicate psychological inflexibility, which shows the tendency to become fused with the content of thoughts and feelings. The measure had excellent internal consistency.^44^ The scale has been validated in Iran. The findings of the study showed that the scale reliability was 0.71;^45^ Cronbach's alpha in this sample was 0.72.

#### Barret Impulsivity Scale

The 11th edition of the Barret Impulsivity Scale (BIS-11^46^) consists of 30 items measuring three subscales: (a) attentional impulsiveness (e.g. ‘I don't pay attention’), (b) motor impulsiveness (e.g. ‘I do things without thinking’) and (c) non-planning impulsiveness (e.g. ‘I plan tasks carefully’). The scale is rated on a 4-point scale (rarely/never, occasionally, often, almost always/always). The scale has good internal consistency and test–retest reliability,^47,48^ with alphas ranging from 0.71 to 0.83. Also, in Iran the scale has been validated, and the findings demonstrated that Cronbach's alpha was 0.81.^49^ In this sample, Cronbach's alpha was 0.84.

#### Childhood Trauma Questionnaire

The Childhood Trauma Questionnaire (CTQ^50^) is a self-report questionnaire with 28 items assessing exposure to a range of childhood traumas. The scale produces five subscales, each with five items. The subscales include: (a) emotional abuse (e.g. ‘I felt that someone in my family hated me’), (b) physical abuse (e.g. ‘I believe that I was physically abused’), (c) sexual abuse (e.g. ‘Someone molested me’), (d) emotional neglect (e.g. ‘People in my family felt close to each other’) and (e) physical neglect (e.g. ‘I had to wear dirty clothes’). Items are rated on a 5-point scale ranging from ‘never true’^1^ to ‘very often true’ in regard to the endorsed frequency of the event,^6^ and the mean scores for each subscale were calculated. The questionnaire also has three items that assess denial that are not used in the analyses. The scales showed moderate to high internal consistency and test–retest correlations.^50^ The findings of a study in Iran which addressed the validation of this measure showed that the scale reliability was good.^51^ Cronbach's alpha reliability of the scales was high in this sample, (α = 0.89).

#### Inventory of Statements About Self-Injury

The Inventory of Statements About Self-Injury (ISAS^52^) is a measure with two behavioural and functional sections that assess lifetime frequency and functions of NSSI behaviours respectively. In Section 1, participants responded to questions regarding NSSI behaviour that indicate the method used. The 12 methods represented in this section are: (a) banging/hitting, (b) biting, (c) burning, (d) carving, (e) cutting, (f) wound picking, (g) needle-sticking, (h) pinching, (i) hair pulling, (j) rubbing skin against rough surfaces, (k) severe scratching and (l) swallowing dangerous substances. The participants indicated the approximate number of times they had performed each behaviour (e.g. 0, 10, 20 times) for each behaviour. The behavioural scales demonstrated good reliability and validity.^53^ In Iran the scale has been validated, and according to the findings, the scale reliability was 0.94.^54^ The Cronbach's alpha reliability of the scales was 0.87 in this sample. The total number of lifetime NSSI was obtained by summing the number of acts across all methods. In addition, the statements in the functional section were rated on a 3-point scale: 0 (not relevant), 1 (somewhat relevant) and 2 (very relevant). In this study we utilised the total number of NSSI behaviours reported, as well as the intrapersonal functions.

### Data analysis

The data underwent analysis utilising the statistical software packages SPSS 26.0 for Windows (SPSS Inc., Chicago, Illinois, USA) and AMOS 26.0 for Windows (Chicago, USA) . Prior to conducting path analysis, we conducted several preliminary assessments to ensure the validity of our data. These assessments included examining the normality of the variables using skewness and kurtosis, with values falling within the acceptable range of ±3. Additionally, we assessed the linearity of the relationships through scatterplot analysis, ensuring that the relationships between variables were linear. We also checked for the absence of collinearity by examining the variance inflation factor (VIF), with values below 7 indicating a lack of collinearity. Furthermore, we assessed the presence of multivariate outliers by calculating Mahalanobis distances and confirmed that no outliers were present. Overall, all assumptions necessary for conducting path analysis were satisfied.

Path analysis was employed to examine the relationships between NSSI and its associated functions as criterion variables, with experiential avoidance as the first mediator and rumination as the second mediator, i.e. the effect initially flows from predictors to experiential avoidance, subsequently passing through rumination and ultimately culminating in the criterion variables. The predictors were categorised into three groups: distress tolerance, interpersonal schemas (referred to as emotional deprivation, abandonment, mistrust/abuse, social isolation, dependence/incompetence, enmeshment/undeveloped self, subjugation, self-sacrifice, emotional inhibition, entitlement/grandiosity) and intrapersonal schemas (referred to as defectiveness/shame, failure, unrelenting standards, insufficient self-control). We specified links between distress tolerance and the mediators and all three criterion variables. Also, we considered interpersonal schemas only to be linked with NSSI and its intrapersonal function, with paths to other variables constrained to zero. Similarly, we considered intrapersonal schemas only to be linked with NSSI and its intrapersonal functions. In each case, we took into account the connection between the predictors and the outcomes, which is mediated by rumination. Specifically, the pathway is as follows: predictor -> experiential avoidance -> rumination -> criterion variables. Additionally, we accounted for impulsivity and childhood maltreatment in our model.

For overall model evaluation, standard model fit criteria were utilised as follows to indicate appropriate model fit: comparative fit index (CFI) > 0.90, Tucker-Lewis index (TLI) > 0.90, root mean square error of approximation (RMSEA) < 0.08, X^2^/d.f. > 2.0, goodness of fit index (GFI) > 0.90, normed fit index (NFI) > 0.90 and standardised root mean squared residual (SRMR) < 0.05. Model paths were evaluated by examining significance and reporting beta coefficient (β). Indirect effects were tested using the bootstrapping function in AMOS with 5000 samples to generate an estimate of indirect effects along with a 95% CI. Significance values for indirect effects were generated by AMOS and can be verified by the presence of 95% CI value ranges that exclude zero.

## Results

### Preliminary analysis

Mean scores, standard deviations, skewness and kurtosis of the variables are presented in Table 1, and Table 2 indicates the correlation matrix of research variables. In this sample, the mean score of NSSI was 4.53, and the standard deviation of NSSI was 4.57. The frequency of NSSI was presented according to the following range: 0–4 per method, where a score of 0 represented never having engaged in that method, a score of 1 represented having engaged in that method 10 times, a score of 2 represented having engaged in that method 100 times, a score of 3 represented having engaged in that method 500 times and a score of 4 indicated having engaged in that method more than 500 times. In addition, banging (53.9%), biting (38.1%) and cutting (35.1%) were the most frequently endorsed in this sample. Before conducting path analysis, we examined normality, linearity of the relationships, lack of collinearity and lack of multivariate outliers, and all assumptions were met. The fit indices for the examined model were good: X^2^/d.f. = 2.25, GFI = 0.98, NFI = 0.97, CFI = 0.98, RMSEA = 0.054, SRMR = 0.028. Given the high number of paths, only significant paths are reported, and the non-significant paths are presented in supplements. As seen in Table 3, social isolation and insufficient self-control were positively and distress tolerance was negatively associated with experiential avoidance. Moreover, vulnerability to harm, emotional deprivation, social isolation and insufficient self-control were positively associated with rumination. Impulsivity and CTQ as the control variables also were positively associated with NSSI and not its functions. The explained variance of the variables in the model was 26%, 53%, 0.08, 0.03 and 13% for experiential avoidance, rumination, intrapersonal functions, interpersonal functions and NSSI scores.

**Table 1:** Summary of key aspects in introduction

Model of emotion regulation for NSSI	Common point of models	Critique of models	Transdiagnostic factor of NSSI in adolescents
Process model of emotion regulation^6^ Difficulties in emotional regulation model^12^ Emotional Cascade Model^22^ Cognitive emotional model of NSSI^20^	Focus on a trigger or situation that evokes emotions Presence of emotional experience Role of cognitive processes	Non-applicability of all models for community-based samples of adolescents Not addressing the constructs related to childhood experiences like early maladaptive schemas in all models Not addressing the role of transdiagnostic factors and their relationship in development and maintenance of NSSI	Early maladaptive schemas Distress tolerance Experiential avoidance Rumination

NSSI, non-suicidal self-injury.

**Table 2:** Correlation matrix of research variables

	1	2	3	4	5	6	7	8	9	10	11	12	13	14	15	16	17	18	19	20	21	22	23
1	–																						
2	0.59**	–																					
3	−0.27**	−0.23**	–																				
4	0.12**	0.16**	−0.04	–																			
5	0.39**	0.39**	−0.12**	0.29**	–																		
6	0.25**	0.28**	−0.12*	0.23**	0.271**	–																	
7	0.11*	0.10*	−0.11*	0.02	0.03	0.19**	–																
8	0.21**	0.25**	−0.16**	0.04	0.17**	0.29**	0.59**	–															
9	0.30**	0.46**	−0.10*	0.20**	0.26**	0.24**	0.12*	0.14**	–														
10	0.21**	0.24**	−0.28**	0.09	0.18**	0.18**	0.10*	0.14**	0.18**	–													
11	0.30**	0.38**	−0.12**	0.08	0.27**	0.18**	0.15**	0.16**	0.33**	0.25**	–												
12	0.37**	0.52**	−0.08	0.19**	0.30**	0.25**	0.15**	0.20**	0.47**	0.18**	0.46**	–											
13	0.35**	0.48**	−0.16**	0.21**	0.34**	0.23**	0.12**	0.24**	0.43**	0.26**	0.35**	0.60**	–										
14	0.37**	0.42**	−0.11*	0.22**	0.39**	0.18**	0.07	0.17**	0.31**	0.18**	0.32**	0.44**	0.58**	–									
15	0.27**	0.34**	−0.10*	0.15**	0.31**	0.12**	0.08	0.19**	0.30**	0.20**	0.22**	0.34**	0.39**	0.52**	–								
16	0.33**	0.43**	−0.26**	0.17**	0.28**	0.21**	0.10*	0.21**	0.32**	0.25**	0.37**	0.41**	0.35**	0.35**	0.36**	–							
17	0.16**	0.23**	−0.19**	0.01	0.16**	0.04	0.10*	0.15**	0.26**	0.21**	0.21**	0.21**	0.22**	0.21**	0.29**	0.25**	–						
18	0.30**	0.38**	−0.17**	0.15**	0.31**	0.15**	0.14**	0.20**	0.31**	0.26**	0.30**	0.41**	0.50**	0.53**	0.49**	0.42**	0.34**	–					
19	0.17**	0.33**	−0.02	−0.03	0.09	0.15**	0.13**	0.21**	0.25**	0.27**	0.28**	0.22**	0.19**	0.12*	0.12**	0.22**	0.23**	0.27**	–				
20	0.43**	0.47**	−0.16**	0.15**	0.30**	0.22**	0.13**	0.14**	0.39**	0.14**	0.34**	0.54**	0.42**	0.36**	0.29**	0.36**	0.25**	0.45**	0.23**	–			
21	0.20**	0.36**	−0.09	−0.07	0.01	0.12**	0.11*	0.11*	0.26**	0.09**	0.26**	0.28**	0.17**	0.07	0.05	0.26**	0.19**	0.11*	0.37**	0.37**	–		
22	0.42**	0.49**	−0.14**	0.10*	0.36**	0.28**	0.14**	0.17**	0.34**	0.23**	0.44**	0.40**	0.29**	0.24**	0.23**	0.31**	0.15**	0.30**	0.39**	0.43**	0.41**	–	
23	0.35**	0.47**	−0.18**	0.12**	0.41**	0.19**	0.15**	0.21**	0.38**	0.22**	0.30**	0.34**	0.40**	0.38**	0.28**	0.32**	0.24**	0.42**	0.29**	0.45**	0.32**	0.44**	–

Notes: 1, AFQ; 2, RRS; 3, distress tolerance; 4, CTQ; 5, BIS; 6, NSSI; 7, interpersonal function; 8, intrapersonal function; 9, emotional deprivation; 10, abandonment; 11, mistrust/abuse; 12, social isolation; 13, defectiveness/shame; 14, failure; 15, dependence/incompetence; 16, vulnerability to harm; 17, enmeshment/undeveloped self; 18, subjugation; 19, self-sacrifice; 20, emotional inhibition; 21, unrelenting standards; 22, entitlement/grandiosity; 23, insufficient self-control.

AFQ, Avoidance and Fusion Questionnaire; RRS, Ruminative Response Scale; CTQ, Childhood Traumatic Experiences; BIS, Barret Impulsivity Scale; NSSI, non-suicidal self-injury.

* Correlation is significant at the 0.01 level (2-tailed).

** Correlation is significant at the 0.05 level (2-tailed).

**Table 3:** Direct effects between variables

Independent variable	Dependent variable	β	*t*	s.e.	*P*
Social isolation	AFQ	0.150	2.57	0.058	<0.010
Insufficient self-control	AFQ	0.152	3.07	0.049	<0.010
Distress tolerance	AFQ	−0.165	−3.68	0.045	<0.001
AFQ	RRS	0.348	9.03	0.039	<0.001
Vulnerability to harm	RRS	0.103	2.55	0.040	<0.050
Emotional deprivation	RRS	0.127	3.19	0.040	<0.010
Social isolation	RRS	0.172	3.68	0.047	<0.001
Insufficient self-control	RRS	0.158	3.98	0.040	<0.001
BIS	NSSI	0.125	2.51	0.050	<0.050
CTQ	NSSI	0.157	3.29	0.047	<0.001
RRS	NSSI	0.134	2.25	0.059	<0.050
RRS	Intrapersonal function	0.157	2.57	0.061	<0.050

Notes: AFQ, Avoidance and Fusion Questionnaire; RRS,  Ruminative Response Scale; BIS, Barret Impulsivity Scale; CTQ, Childhood Traumatic Experiences; NSSI, non-suicidal self-injury.

### Tests of mediating effects

**Fig. 1:**
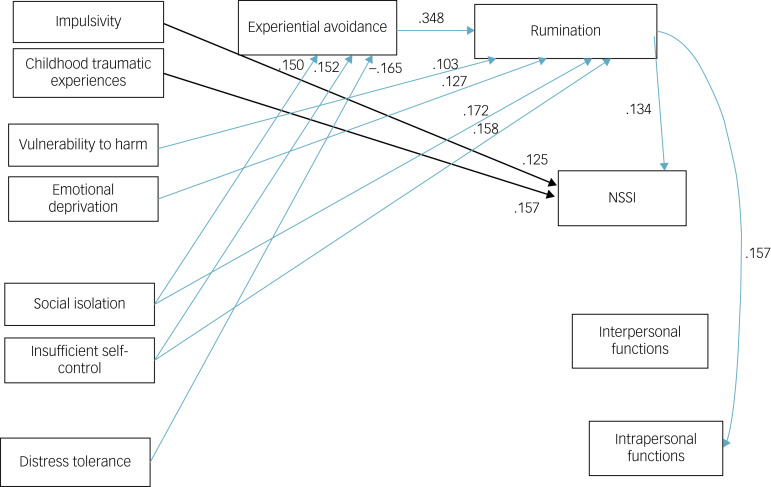
Final model of direct and indirect paths. NSSI, non-suicidal self-injury.

The results of mediation effects are presented in Table 4. According to the results, some of the paths in the first model were not significant, but in the final model, all the reported path coefficients were found to be significant at least at the *P* < 0.05 level. As seen in Table 4, rumination significantly mediated the relationship between vulnerability to harm, emotional deprivation, social isolation, insufficient self-control, and NSSI and intrapersonal function. Fig. 1 shows the direct and indirect path among variables with beta coefficient for each of the path. As seen in Fig. 1, in serial fashion, experiential avoidance mediated the role of rumination in the relationship between social isolation, and insufficient self-control, and NSSI and intrapersonal function. All indirect effects were significant. Finally, the findings also showed that experiential avoidance and rumination acted as mediators in a sequence in the relationship between distress tolerance, and NSSI and intrapersonal function. The beta coefficient (β), SE and indirect effects for each path are reported in Table 4.

**Table 4:** Indirect effects and 95% confidence interval (CI)

Indirect effect	β	s.e.	95% CI		*P*	β
			Lower	Upper		
Vulnerability to harm → RRS → NSSI	0.014	0.009	0.003	0.032	0.027	0.014*
Vulnerability to harm → RRS → Intrapersonal function	0.016	0.009	0.004	0.037	0.011	0.016*
Emotional deprivation → RRS → NSSI	0.017	0.010	0.005	0.042	0.019	0.017*
Emotional deprivation → RRS → Intrapersonal function	0.020	0.010	0.007	0.042	0.006	0.020**
Social isolation → AFQ → RRS	0.052	0.023	0.014	0.089	0.016	0.052*
Social isolation → AFQ → RRS → NSSI	0.007	0.005	0.001	0.017	0.032	0.052*
Social isolation → AFQ → RRS → Intrapersonal function	0.008	0.005	0.002	0.019	0.012	0.052*
Social isolation → RRS → NSSI	0.023	0.013	0.006	0.050	0.026	0.023*
Social isolation → RRS → Intrapersonal function	0.027	0.014	0.009	0.056	0.007	0.027**
Insufficient self-control → AFQ → RRS	0.053	0.021	0.021	0.090	0.006	0.053**
Insufficient self-control → AFQ → RRS → NSSI	0.007	0.005	0.002	0.018	0.023	0.053*
Insufficient self-control → AFQ → RRS → Intrapersonal function	0.008	0.005	0.002	0.019	0.010	0.053**
Insufficient self-control → RRS → NSSI	0.021	0.011	0.005	0.044	0.025	0.021*
Insufficient self-control → RRS → Intrapersonal function	0.025	0.012	0.009	0.050	0.007	0.025**
Distress tolerance → AFQ → RRS	−0.058	0.017	−0.089	−0.032	0.001	−0.057***
Distress tolerance → AFQ → RRS → NSSI	−0.008	0.004	−0.018	−0.002	0.021	−0.057*
Distress tolerance → AFQ → RRS → Intrapersonal function	−0.009	0.005	−0.019	−0.003	0.007	−0.057**
AFQ → RRS → NSSI	0.047	0.023	0.010	0.087	0.038	0.047*
AFQ → RRS → Intrapersonal function	0.054	0.024	0.019	0.097	0.011	0.055*

AFQ, Avoidance and Fusion Questionnaire; RRS, Ruminative Response Scale; BIS, Barret Impulsivity Scale; CTQ, Childhood Traumatic Experiences.

***P < 0.001, **P < 0.010, *P < 0.050.

## Discussion

The purpose of this study was to investigate the relationship between EMS and NSSI and its functions, and the link between distress tolerance and NSSI and its functions. The study also examined the potential mediating effects of experiential avoidance and rumination on the EMS–NSSI association as well as distress tolerance–NSSI association. Furthermore, impulsivity and childhood traumatic experiences were controlled in this study.

As expected, the results indicated that rumination predicted NSSI. This finding is consistent with a large body of research.^19^ Regarding indirect effects, the findings indicated that rumination mediated the relationship between schemas including vulnerability to harm, emotional deprivation, social isolation, insufficient self-control and NSSI. Previous researchers have indicated that these schemas are predictors of depressive symptoms and rumination.^29^ Finally, as a result, an increase in depressive components of rumination can lead to NSSI engagement. In fact, the path follows a sequence from schema activation which leads to some responses, of which one is rumination.^29^ Ruminative response is difficult to manage for adolescents, so as a way of coping with this process, the adolescent uses NSSI. The results also showed that the relationship between the schemas of self-control and social isolation and NSSI is sequentially mediated by experiential avoidance and rumination. On the one hand, this finding suggests that the mentioned schemas lead to experiential avoidance, then experiential avoidance promotes rumination, which in turn causes NSSI engagement. Individuals with insufficient self-control schema, which is related to difficulty in exercise of self-control and frustration tolerance to achieve goals, cannot tolerate aversive experiences.

On the other hand, it has been shown that the social isolation schema has been related to shame,^55^ and it can explain how adolescents with this schema have a feeling of being isolated; shame can be evoked in some particular situations, and experiential avoidance here serves as an escape. In addition, it is very important to consider that the role of shame can be triggered by many parameters in this developmental stage,^56^ that can be specific in adolescence rather than at other developmental stages. So, it seems that among the schemas, these two schemas lead the adolescents to avoid internal experiences evoked by the schema. Furthermore, based on the experiential avoidance conceptualisation of depressive rumination,^30^ experiential avoidance leads to ruminative thinking, and this path ends in NSSI engagement. In fact, it seems that after activation of the schemas, the individual experiences different aversive emotions and tries to avoid them and as a result uses some coping mechanisms like experiential avoidance, which leads to avoiding aversive emotions that are difficult.^25^ However, this is not the end of the process, because there are also ruminative thoughts in the sequence that are difficult to manage, so the individual tries to use NSSI as a way of ending this whole sequence. However, this path from schemas, experiential avoidance and rumination to NSSI, is weak compared with direct effects. An explanation for this might be the fact that the potential effects of other variables, like comorbid disorders, can be considered as confounding variables, because it may be that the presence of these variables leads to other potential paths.

The results showed that there is an indirect path between distress tolerance and the intrapersonal function of NSSI, mediated sequentially by experiential avoidance and rumination. Previous research has shown that distress tolerance is associated with experiential avoidance,^57^ so it may be that individuals with low distress tolerance try to avoid their aversive experiences, and based on the experiential avoidance conceptualisation of rumination,^30^ following the avoidance of emotions, the individual with low distress tolerance employs ruminative thinking which in turn leads to the use of NSSI as a means of regulating internal experiences (the intrapersonal function of NSSI). In addition, the findings interestingly showed that the same schemas (vulnerability to harm, emotional deprivation, social isolation and insufficient self-control) that were linked to NSSI via rumination were also associated with intrapersonal functions of NSSI, through rumination. The link between theses schemas and an increase in depressive symptoms and rumination was discussed above; then, an increase in rumination leads to the use of NSSI as a function of regulating one's emotions (general intrapersonal function of NSSI), which is consistent with the findings of the study conducted by Quirk and Wier.^29^ With regard to the schemas of emotional deprivation and social isolation that were considered to be interpersonal schemas, the findings suggested that they were linked to the intrapersonal function of NSSI, a finding that was not expected. Since both of these schemas belong to the domain of disconnection and rejection, and since individuals with these schemas are sensitive to rejection in their relationships and do not have enough interpersonal skills, it may be that they use NSSI as a way of regulating themselves after the rejection. The results indicated that the schemas of insufficient self-control and social isolation were linked to intrapersonal functions of NSSI via experiential avoidance and rumination. The relationship in the path between schemas and experiential avoidance and rumination was discussed above. With regard to the relationship between rumination and the intrapersonal function of NSSI, consistent with previous research by Quirk and Wier,^29^ more ruminative thinking can lead the individuals to use NSSI as a coping strategy for emotion regulation. With regard to the interpersonal functions of NSSI, the results did not show any significant direct or indirect effect. The previous research also indicated that adolescents who engage in NSSI mainly use this behaviour for intrapersonal reasons;^58^ this is consistent with the fact that the main function of the NSSI is emotion regulation^1^, which is considered to be an intrapersonal function of NSSI. In addition, we should consider cultural and developmental aspects. For example, it might be that for Iranian adolescents, like other Asian adolescents,^59^ who are involved with both collectivist and individualistic values of both Asian and Western culture, the intrapersonal function of NSSI can be considered as a way of coping with stress derived from that diversity in cultures.

### Clinical implications

Several clinical implications and suggestions arise from the present research. First, working with patients on understanding unique patient elements of EMS can help them understand deeply ingrained, maladaptive self-beliefs that result in highly self-critical and self-punishing behaviour, including NSSI. Addressing maladaptive EMS beliefs can also impact how individuals perceive themselves in relationships and how they interact with others. Given that many who self-injure report interpersonal factors in driving NSSI, in addressing EMS individuals can develop healthier relationship dynamics and communication patterns. Likewise, EMS influence thought patterns that are likely to aggravate ruminative responses, which increase emotional sensitivity and reactivity, a major factor in NSSI behaviour. Reducing EMS will likely lead to reductions in rumination, which could have several positive downstream effects. Furthermore, by learning to address rumination and challenge negative self-schemas, individuals can develop better emotional regulation skills, leading to more stable and balanced mood states. Finally, helping patients address both rumination and early maladaptive self-schemas can empower individuals to take control of their thoughts, emotions and behaviours, leading to a greater sense of agency and self-efficacy.

### Limitations and future directions

The study had some limitations, which can be divided into sampling issues and measurement issues. Regarding sampling issues, the first limitation was that the sample was limited to the adolescents of some selected schools, and results may have changed if more schools had participated. Second, although this was a community sample, it was not a treatment-seeking sample; accordingly, the generalisabity of the findings to clinical samples, especially those with comorbid disorders, should be interpreted with caution. With regard to measurement issues, the third limitation was that all the measures used were self-report scales, which could raise concerns about recall bias and desirability. Fourth, it might be that the potential presence and role of some confounding variables like other mental disorders and personality disorders affected the results, since these variables were not considered in this study. Fifth, given that the individuals who took part in this study were adolescents from a convenient sample, the findings may not be representative. In fact, the findings may not be applicable for the general population. In addition, since the study was cross-sectional, the potential mediational and causal effects suggested in this study should be verified with longitudinal designs. Regarding adolescent populations, it is likely important for future work to use interviews in addition to self-report measures, so that self-injurious behaviours could be more clearly and accurately reported than is possible with self-report alone; in order to decrease recall bias of self-report measures, interviews, behavioural tasks and ecological momentary assessment could be used. In addition, it is important to consider the role of important confounding variables, like other mental disorders and personality disorders, and to acknowledge that the magnitude of some indirect effects was relatively small compared with direct effects. Last, other transdiagnostic and cognitive factors should be examined to determine their role in NSSI engagement and functions; it is suggested that future studies should address other factors that could be considered as potential serial mediators using the same framework in this study.

## Conclusion

The current study presented a model of transdiagnostic factors in adolescents with NSSI. The main findings of the study revealed that there is a link between the schemas of social isolation, insufficient self-control, and NSSI and the intrapersonal function of NSSI; also, the association between distress tolerance, and NSSI and the intrapersonal function of NSSI is sequentially mediated by experiential avoidance and rumination. This pathway can demonstrate the importance of addressing the transdiagnostic factors of experiential avoidance and rumination in therapeutic interventions for NSSI in adolescents. Furthermore, the findings of the study also suggest the importance of targeting the discussed factors in treatment of NSSI. Since the paths between the EMS and distress tolerance with NSSI were mediated by experiential avoidance and rumination, it is important to target these factors in therapeutic interventions.

## Data Availability

The data that support the findings of this study are available from the corresponding author upon reasonable request.
